# Cost-Effectiveness of Internet-Based Self-Management Compared with Usual Care in Asthma

**DOI:** 10.1371/journal.pone.0027108

**Published:** 2011-11-11

**Authors:** Victor van der Meer, Wilbert B. van den Hout, Moira J. Bakker, Klaus F. Rabe, Peter J. Sterk, Willem J. J. Assendelft, Job Kievit, Jacob K. Sont, on behalf of the SMASHING (Self-Management in Asthma Supported by Hospitals, ICT, Nurses and General Practitioners) Study Group

**Affiliations:** 1 Department of Medical Decision Making, Leiden University Medical Center, Leiden, The Netherlands; 2 Department of Public Health and Primary Care, Leiden University Medical Center, Leiden, The Netherlands; 3 Department of Pulmonology, Leiden University Medical Center, Leiden, The Netherlands; 4 Department of Respiratory Medicine, Academic Medical Center, Amsterdam, The Netherlands; University of Liverpool, United Kingdom

## Abstract

**Background:**

Effectiveness of Internet-based self-management in patients with asthma has been shown, but its cost-effectiveness is unknown. We conducted a cost-effectiveness analysis of Internet-based asthma self-management compared with usual care.

**Methodology and Principal Findings:**

Cost-effectiveness analysis alongside a randomized controlled trial, with 12 months follow-up. Patients were aged 18 to 50 year and had physician diagnosed asthma. The Internet-based self-management program involved weekly on-line monitoring of asthma control with self-treatment advice, remote Web communications, and Internet-based information. We determined quality adjusted life years (QALYs) as measured by the EuroQol-5D and costs for health care use and absenteeism. We performed a detailed cost price analysis for the primary intervention. QALYs did not statistically significantly differ between the Internet group and usual care: difference 0.024 (95% CI, −0.016 to 0.065). Costs of the Internet-based intervention were $254 (95% CI, $243 to $265) during the period of 1 year. From a societal perspective, the cost difference was $641 (95% CI, $−1957 to $3240). From a health care perspective, the cost difference was $37 (95% CI, $−874 to $950). At a willingness-to-pay of $50000 per QALY, the probability that Internet-based self-management was cost-effective compared to usual care was 62% and 82% from a societal and health care perspective, respectively.

**Conclusions:**

Internet-based self-management of asthma can be as effective as current asthma care and costs are similar.

**Trial Registration:**

Current Controlled Trials ISRCTN79864465

## Introduction

Asthma is a chronic, inflammatory disorder of the airways clinically characterized by respiratory symptoms such as wheeze, cough, dyspnoea, chest tightness and impaired lung function [1], [2]. Treatment for asthma is aimed at improving asthma control, i.e. reducing current symptoms and need for short-acting bronchodilation, improving lung function and preventing future exacerbations [1]–[3].

In the past decade, the care for asthma patients has shifted from physician-managed care to guided self-management. Guided self-management includes asthma education, self-monitoring of symptoms and/or lung function and adjustment of treatment according to an action plan guided by a health care professional (not necessarily a physician). Self-management has been shown to improve asthma control and quality of life and reduce health care utilization and sometimes improve lung function [4].

Besides clinical effectiveness, the implementation of new disease management strategies requires an economic evaluation to determine whether the clinical benefits are gained at reasonable costs. Several cost evaluations have compared paper-and-pencil self-management plans to usual care in asthma [5]–[11], but only a few compared costs to quality of life [10]–[11]. Most of these economic evaluations found that written self-management plans for asthma were likely to be cost-effective compared to usual physician provided care. However, the implementation of paper-and-pencil self-management plans is hampered by patients' and doctors' reluctance to use written diaries [12].

Implementation of guided self-management programs may be enhanced by the use of Internet-based technologies, particularly in remote and underserved areas. In a recently conducted randomized controlled trial we have shown that Internet-based self-management is feasible and provides better clinical outcomes compared to usual physician provided care with regard to asthma related quality of life, asthma control, symptom-free days and lung function [13]. Although previous trials have also evaluated the clinical effects of Internet-based self-management in adults [14] and children [15], [16], so far, no economic evaluations have been conducted. We therefore carried out a cost-utility analysis, comparing quality of life with societal and health care costs during one year, to determine whether the clinical benefits gained with Internet-based self-management are attained at reasonable costs.

## Methods

The protocol for this trial and supporting CONSORT checklist are available as supporting information; see Protocol S1, Checklist S1, and Flowchart S1.

### Ethics statement

The study was approved by the Medical Ethics Committee of the Leiden University Medical Center. All participants gave their written consent.

### Setting and Participants

Two hundred patients participated in a 12-month multicenter, non-blinded, randomized controlled trial. Patients were recruited from 37 general practices (69 General Practitioners) in the Leiden and The Hague area and the Outpatient Clinic of the Department of Pulmonology at the Leiden University Medical Center, The Netherlands over the period from September 2005 to September 2006 [13]. We included patients with physician diagnosed asthma as coded according to the International Classification of Primary Care in the electronic medical record [17], aged 18–50 years, with a prescription of inhaled corticosteroids for at least three months in the previous year, access to Internet at home, mastery of the Dutch language and without serious comorbid conditions that interfered with asthma treatment. Patients on maintenance oral glucocorticosteroid treatment were excluded. All participants gave their written consent.

Details of the randomization and intervention have been described previously [13]. Briefly, the 200 patients were randomly assigned to Internet-based self-management as an adjunct to usual care (Internet group: 101 patients) or to usual physician-provided care alone (usual care group: 99 patients). Allocation took place by computer after collection of the baseline data, ensuring concealment of allocation. The Internet-based self-management program included weekly monitoring of asthma control and lung function, immediate treatment advice according to a computerized personal action plan after completing the validated Asthma Control Questionnaire on the Internet [18], on-line education and group-based education, and remote Web communication with a specialized asthma nurse.

### Utilities and QALYs

Utilities express the valuation of health-related quality of life on a scale from zero (death) to one (perfect health). Patients described their health-related quality of life using the EuroQol classification system (EQ-5D) [19], from which we calculated their utilities over time using the British tariff [20]. The area under the utility curve is known as quality-adjusted life years (QALY) and was used as the primary outcome measure for the cost-effectiveness analysis. Patients additionally valued their own health status on a visual analogue scale (VAS). This scale from the patient perspective is potentially more responsive to change than other generic quality of life instruments, but is not the best choice for economic evaluations from a societal perspective [21]. The VAS scale was transformed to a utility scale using the power transformation 1−(1-VAS/100)^1.61^ [22].

We obtained utility measurements at baseline, 3 and 12 months. For EQ-5D measurements 6.5%, 10% and 8.5% were missing and for visual analogue measurements 7%, 10% and 9% were missing at 0, 3 and 12 months, respectively. To correct for possibly selective non-response, missing measurements were replaced by 5 imputed values based on switching regression [23], [24] with regression variables randomisation group, age, sex, asthma control at baseline and available utility measures at all time points.

### Costs

We distinguished three major cost categories: intervention costs, other health care costs and productivity costs [10], [11]. Intervention costs consisted of materials (software support, electronic spirometer), personnel and patient costs (travel, time, Internet and text messaging costs). Other health care costs included contacts (including face-to-face, telephonic and home contacts) with health care professionals (general practitioners, chest physician, other specialists, physiotherapists, psychologists, complementary care and other paramedical professionals), emergency room visits, hospital admissions and both asthma and non-asthma medication. Productivity costs consisted of hours of absence from work.

Patients reported their use of health care resources and the hours of absence from work using a quarterly cost-questionnaire. We used Dutch standard prices for units of resource use (contacts with health care professionals, hospital admissions and drug prescriptions) and hours of absenteeism, designed to represent societal costs and to standardize economic evaluations [25], [26]. Hours of absenteeism were converted to costs by multiplying them with the age and gender average hourly wage [25]. Details of the drugs used were derived from pharmacy records. All prices were converted to the price level of 2007 according the general Dutch consumer price index [27] and converted to US dollars using the purchasing power parity index (€1 = $1.131) [28]. Because of the one-year time horizon, costs were not discounted.

Cost-questionnaires were scheduled to be handed in at 3, 6, 9 and 12 months. Of these quarterly questionnaires, 10%, 14%, 19% and 9% were missing, respectively. Pharmacy records were available for 182 patients (91%). Missing cost-questionnaire and pharmacy record were imputed using multiple imputation, as previously described under ‘Utilities and QALYs’.

### Statistical Analysis

Differences and statistical uncertainty of QALYs and costs were calculated using non-parametric bootstrap estimation with 5000 random samples (1000 from each of the 5 imputations). Differences in costs resulted from differences in volumes rather than differences in unit costs, since we used standard prices for units of resource use and hours of absenteeism. We estimated the intervention effect by a linear regression model with randomisation group as only independent variable, combining the 5 multiple imputation sets using Rubin's rules [29].

Analyses were carried out with Stata 9.0 (StataCorp, College Station, TX).

### Cost-Effectiveness Analysis

The base case cost-effectiveness analysis compared societal costs with QALYs gained based on the British EQ-5D over the period of one year. Because of the limited degree of modeling in this cost utility analysis, we carried out sensitivity analyses only on the use of different utility measures (British EQ-5D or Visual Analogue Scale) and on the included cost categories (societal or healthcare perspective).

**Figure 1:**
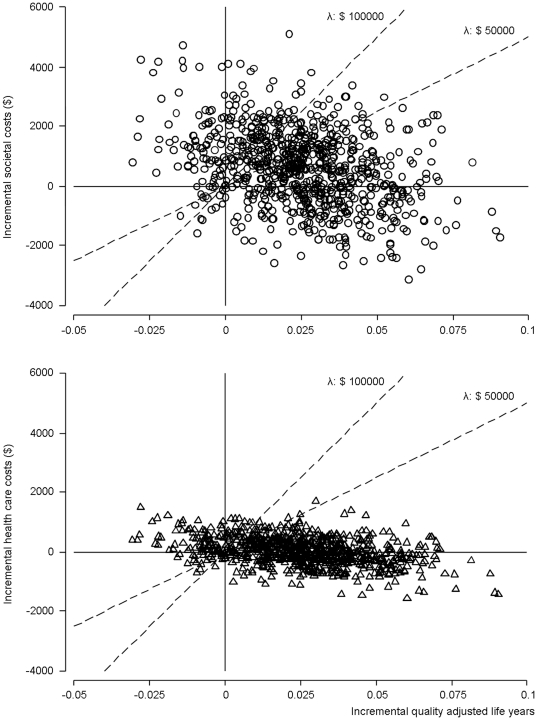
Cost-effectiveness planes. Uncertainty about cost-effectiveness of the asthma internet-based self-management program compared with usual asthma care (showing the 1000 bootstrapped estimates). Circles and triangles represent the incremental societal and health care costs, respectively, plotted against the incremental quality adjusted life years (QALY) (intervention minus usual care). The south-east quadrant indicates that internet-based self-management intervention dominates usual care (i.e. effectiveness is higher and costs are lower), the north-west quadrant indicates that usual care dominates the intervention. The points below the dashed diagonal lines are cost-effective at a willingness to pay threshold of $50000 and $100000 per QALY, respectively.

**Figure 2:**
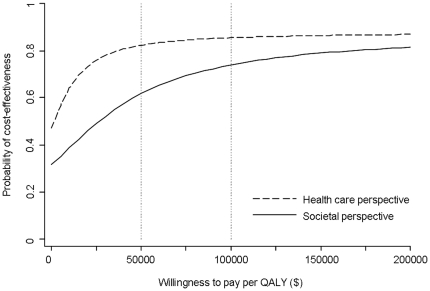
Cost-effectiveness acceptability curves. The probability that Internet-based self-management is cost-effective compared to usual care depending on the willingness-to-pay per QALY from a societal perspective *(solid line)* and health care perspective *(dashed line)*.

Statistical uncertainty of the cost-effectiveness was analyzed using the net benefit approach [30]. The net benefit is defined as λ x ΔQALY – Δcosts, where λ is the willingness to pay for a gain of one quality-adjusted life year. This way, the observed QALY difference is reformulated into a monetary difference. The uncertainty about cost-effectiveness was graphically shown by plotting the bootstrapped incremental costs and QALY estimates in the cost-effectiveness plane (200 estimated pairs for each of the 5 imputed datasets) (figure 1). In a cost-effectiveness acceptability curve we graphed the probability (1−[one sided] P value) that the Internet-based self-management program was cost-effective (i.e. had higher net benefit) compared with usual care, as a function of λ for a range of λ between 0 and 200000 (figure 2). We highlighted this probability at commonly cited willingness-to-pay values of $50000 and $100000 per QALY [31].

## Results

The Internet group and usual care group consisted of 101 and 99 participants, respectively. Mean age of the sample was 37 years and 70% of the participants were women (table 1). At baseline, asthma related quality of life, asthma control and medication use were similar for the two randomization groups.

**Table 1:** Baseline characteristics.

	Usual Care Group(n = 99)	Internet Group(n = 101)
Women,	71%	68%
Age, years	37 (18–50)	36 (19–50)
Asthma duration, years	18 (0–47)	15 (1–47)
Education level		
Low	14%	11%
Middle	33%	37%
High	53%	52%
Care provider		
General practitioner	80%	79%
Chest physician	20%	21%
FEV_1_ (pre-bronchodilator), L	3.13 (1.56–5.23)	3.08 (1.14–5.19)
FEV_1_ (pre-bronchodilator), % predicted	90 (53–118)	88 (34–133)
Inhaled corticosteroid dose, µg/day	517 (0–2000)	494 (0–1000)
Inhaled long-acting β^2^-agonist, % of patients	60%	59%
Leukotriene modifier, % of patients	2%	3%
Clinical outcomes		
Asthma Quality of Life Questionnaire*	5.79 (3.03–7.00)	5.73 (3.66–6.94)
Asthma Control Questionnaire†	1.11 (0–3.86)	1.12 (0.07–3.22)
Patient utilities‡		
EQ-5D utility	0.89 (−0.06–1.00)	0.91 (0.49–1.00)
EQ-5D visual analogue scale	74 (35–100)	73 (20–100)

Data are mean (range) unless otherwise indicated.

*Range 1 (worst) – 7 (best) [19].

† Range 0 (best) – 6 (worst) [18].

‡ EQ-5D = EuroQol questionnaire, 5 dimensions [20]. Parts of this table were published previously [13].

### Utilities and QALYs

At baseline, the utilities according to the EQ-5D did not statistically significantly differ between the Internet group and the usual care group. EQ-5D utilities did not reach a statistical significant difference throughout the study. At 3 months and 12 months the difference in EQ-5D utility was 0.037 (95% CI, −0.007 to 0.081) and 0.006 (95% CI, −0.042 to 0.054), respectively. Similarly, the difference in quality adjusted life years was not statistically significant: 0.024 (95% CI, −0.016 to 0.065) (table 2).

**Table 2:** Utilities at 0, 3 and 12 months and QALYs*.

Variable	Usual Care Group	Internet Group	Difference (95% CI)	P value
EQ-5D				
0 months	0.89	0.91	0.026 (−0.024 to 0.076)	0.31
3 months	0.89	0.93	0.037 (−0.007 to 0.081)	0.099
12 months	0.91	0.92	0.006 (−0.042 to 0.054)	0.80
QALYs	0.90	0.92	0.024 (−0.016 to 0.065)	0.25
Visual analogue scale†				
0 months	0.87	0.86	−0.013 (−0.045 to 0.019)	0.43
3 months	0.87	0.89	0.012 (−0.026 to 0.050)	0.54
12 months	0.88	0.89	0.013 (−0.015 to 0.040)	0.37
QALYs	0.88	0.88	0.007 (−0.017 to 0.032)	0.57

*Values are summary estimates of the 5 data sets obtained by multiple imputation, combined using Rubin's rules.

† Transformed using the power transformation 1−(1-VAS/100)^1.61^ [23].

Visual analogue scale utilities were not statistically significantly different throughout the study. At 3 and 12 months the difference in visual analogue scale utility was 0.012 (95% CI, −0.026 to 0.050) and 0.013 (95% CI, −0.015 to 0.040), respectively. The difference in quality of life years based on the visual analogue scale was estimated to be 0.007 (95% CI, −0.017 to 0.032) (table 2).

### Costs

The total intervention costs were estimated at $25675, which is $254 (95% CI, $243 to $265) per patient (table 3). The highest cost components of the Internet-based intervention were software support ($7917) and the patients' time costs ($5380 for monitoring time and $5106 for attending the education sessions).

**Table 3:** Implementation costs ($) of Internet-based self-management intervention.

Component of cost	Cost per unit	Number of units	Total cost
Materials			
software support	7917/yr	1	7917
electronic spirometer	19.22/device	101	1942
Personnel			
development educational aids	26/hr	16	412
education sessions	26/hr	30	780
data review and patient communication	26/hr	91	2351
Patient costs			
travel costs for sessions	6/session	258	1465
time costs for sessions (incl. travel time)	20/session	258	5106
time costs for monitoring*	0.50/log in	10873	5380
Internet log in costs†	0.0016/log in	9374	15
mobile phone costs‡	0.20/message	1499	305
Total implementation costs			25675
Total implementation costs per patient			254

*Monitoring time was estimated at 3 minutes per log in and valued at $10 per hour, i.e. the Dutch standard price for unpaid labour [27]. Number of units was obtained from Internet log files.

† Internet costs were valued at $23 per month.

‡ Mobile phone costs were valued at $0.20 per message.

The difference in other health care costs was not statistically significant: $-217 (95% CI, $−1117 to $682) (table 4). Patients in the Internet group had fewer contacts with physiotherapists ($−120, p = 0.03), but not with other health care providers, e.g. general practitioners ($−69, p = 0.18). Similarly differences in costs for medication did not reach statistical difference (table 4). The difference in total health care costs was negligible: $37 (95% CI, $−874 to $950).

**Table 4:** Average health care costs and societal costs per patient ($).

	Usual Care Group		Internet Group		Difference	
	Volume	Costs	Volume	Costs	Costs	P Value
Intervention costs	-	-	1	254	254	<0.001
Other health care costs						
General practitioner*	12.2	294	10.0	225	−69	0.18
Chest physician	0.9	63	0.6	42	−21	0.20
Other specialist	2.5	167	2.3	155	−12	0.75
Physiotherapist	8.6	234	4.2	114	−120	0.03
Psychologist	1.1	161	1.2	180	18	0.78
Complementary care	1.4	87	1.2	75	−12	0.66
Other paramed. professionals	1.5	43	0.8	24	−19	0.28
Emergency room	0.3	45	0.2	35	−10	0.47
Day admissions	0.3	92	0.3	86	−6	0.88
Hospitalizations	1.5	589	1.4	571	−17	0.95
Drugs†						
Short-acting β2-agonists	54%	28	50%	20	−8	0.26
Inhaled corticosteroids (ICS)	50%	89	52%	77	−12	0.47
Long-acting β2-agonists (LABA)	10%	26	11%	20	−6	0.67
Combination ICS+LABA	55%	264	71%	345	82	0.09
Leukotriene antagonists	8%	21	23%	46	25	0.12
Oral corticosteroids	12%	2	13%	2	−1	0.50
Non-asthma medication	99%	312	97%	285	−27	0.71
Subtotal other health care costs		2518		2300	−217	0.63
Total health care costs		2518		2555	37	0.94
Productivity costs‡	98 hr	3131	114 hr	3735	604	0.56
Total societal costs		5647		6289	641	0.63

*General practitioner costs consist of telephonic contacts, office visits and home visits.

† Volumes of drugs represent percentage of patients.

‡ Volumes of productivity costs are number of hours of absence from work.

Patients in the Internet group reported 114 hours of absence from work compared to 98 hours for patients in the usual care group. The 16 hours difference in absenteeism was estimated to be equivalent to $604 (95% CI, $−1430 to $2637) in monetary terms. The difference in societal costs (i.e. health care costs plus costs due to absenteeism) was therefore estimated at $641 (95% CI, $−1957 to $3240) in favor of usual care.

### Cost-utility analysis

The estimates of the cost differences and QALY differences were both not-statistically significant. The cost-utility ratio, based on these point estimates, was $26700 per QALY. The probability that Internet-based self-management was both more effective and less costly than usual care (dominant) was 30%. The probability that it was less effective, but more costly (dominated) was 10% (figure 1). Due to statistical uncertainty of both costs and QALYs, the probability that Internet-based self-management is cost-effective compared to usual care depends on the willingness-to-pay per QALY. This probability was 62% at $50000 per QALY and 74% at $100000 per QALY (figure 1 and 2).

From a health care perspective, the lower health care costs result in a cost-utility ratio of $1500 per QALY. The probability that Internet-based self-management is cost-effective from a health care perspective was 82% at $50000 per QALY and 86% at $100000 per QALY (figure 1 and 2).

QALYs gained, based on the visual analogue scale, were less than those based on the EQ-5D. The probability that Internet-based self-management is cost-effective based on visual analogue scale QALYs was 49% and 60% at $50000 and $100000 per QALY from a societal perspective and was 71% and 75% at $50000 and $100000 per QALY from a health care respectively.

## Discussion

In this study we evaluated the cost-effectiveness of a new disease management strategy, Internet-based self-management, for patients with asthma. The QALY and cost differences, 0.024 and $ 641 respectively, between Internet based-self management and usual care were not statistically significant during a follow-up period of 1 year. Both the estimation of QALYs gained and the calculated expenses showed considerable uncertainty, which is displayed by the cost-effectiveness planes. The estimated cost-utility ratio was $26700 per QALY, which is generally considered acceptable [32]. At a commonly cited willingness-to-pay threshold of $50000 per QALY [31] the Internet-based self-management intervention had a probability of 62% and 82% to be cost-effective compared to usual care from a societal perspective and health care perspective, respectively.

We have previously shown substantial and statistically significant clinical effects in favor of Internet-based self-management with regard to asthma related quality of life, asthma control and lung function [13], [33]. Although the utility outcomes presented in the current study point in the same direction (i.e. in favor of Internet-based self-management) as the clinical outcomes, their statistical significance is less evident. There are two main reasons that may explain this finding. First, generic quality of life measures, such as the EQ-5D, must be distinguished from disease-specific quality of life measures, such as the Asthma Quality of Life Questionnaire [33]. The latter is well known to be responsive to change [21]. However, generic preference-based instruments may differentiate between the highest en lowest levels of asthma control, but are less able to discriminate between moderate levels [34], [35]. The baseline asthma control scores found in our primary care study population can be classified as moderately or partly controlled asthma and substantial improvements in disease-specific quality of life may have been missed by the generic instruments. Second, the absence of a statistically significant difference in our primary utility measure may reflect a lack of statistical power, since our trial was powered to detect a statistical difference in the primary outcome measure, asthma related quality of life, and not explicitly to detect differences in generic preference-based utility measures [13], [36].

The intervention costs of $254 per patient were similar to intervention costs of a paper-and-pencil asthma self-management program [10], but were half of the costs of intensive nurse-led telemonitoring in asthma reported by others [11]. The costs of the technological innovation (software support, electronic spirometer, Internet and mobile phone costs) were only about 40% of the total intervention costs. The fixed technological costs of software support constituted about one third of the intervention costs, so a considerable increase in the number of users could reduce the cost per user by one third. Moreover, the calculations were based on costs during the one-year randomized controlled trial. Asthma self-management cost-effectiveness studies with a longer time horizon have shown that intervention costs decrease after the first year [10], [37]. In our study, costs for education sessions only apply to the first year, thus reducing costs in later years by about a quarter.

Differences in other health care costs should be interpreted with caution, since almost all components showed statistically non-significant differences. Only the reduction in contacts with physiotherapists were statistically significant, suggesting that patients in the Internet group with better asthma control are less in need for physiotherapy. The cost of drugs for asthma show small decreases in short-acting β2-agonists and inhaled corticosteroids alone, but increases in combination therapy (inhaled corticosteroids plus long-acting β2-agonists) and leukotriene antagonists in the self-management group. The increase in volumes and costs of asthma controller medication accompanied by a decrease in reliever medication might have contributed to improved clinical outcomes in favor of Internet-based self-management.

Our study had several limitations. First, quality adjusted life year estimates were calculated from only two follow-up measurements. More measurements would possibly have resulted in more accurate QALY estimates, but we limited the number of follow-up measures in order to minimize the awareness of participating in a clinical trial among patients in the usual care group. Second, patients were inevitably aware of the allocated group, which may have influenced their utility ratings. Therefore, the effects observed may be due to unblinding. On the other hand, the influence of unblinded groups in pragmatic trials might be regarded as part of the intervention, since all interventions implemented in daily clinical practice are not blinded. Third, our economic evaluation was limited to one year. As pointed out above a longer duration would probably have resulted in reduced intervention cost estimates after one year. It is, however, unknown how EQ-5D utility scores will progress after one year.

New cost-effective disease management strategies for asthma are required to face up to the global burden of asthma. Internet-based self-management is an innovative and effective management strategy in adults with asthma that improves clinical outcomes [13]. This Internet-based strategy can be as effective as current asthma care with regard to quality of life and costs are similar. Future implementation studies ought to add other quality of life measures in order to reveal potentially more subtle differences.

## Supporting Information

Protocol S1 Trial protocol
(DOC)

Checklist S1 CONSORT checklist
(DOC)

Flowchart S1 CONSORT flowchart
(TIF)
