# Supplementary material for: Cost-Effectiveness of Internet-Based Self-Management Compared with Usual Care in Asthma
Source: PLoS One. 2011 Nov 11;6(11):e27108. doi: 10.1371/journal.pone.0027108 (PMC3214043; doi:10.1371/journal.pone.0027108)
Supplement: Protocol S1 — Trial protocol (DOC) [file pone.0027108.s001.doc]

**SMASHING in adults**

**Self-Management of Asthma Supported by Hospitals, Information and communication technology, Nurses and General practitioners**

**Protocolnummer: P05.136**

**versie 1.1**

**31 oktober 2005**

Verrichter/opdrachtgever: dr JK Sont, epidemioloog afdeling medische besliskunde LUMC

Uitvoerders: dr JK Sont, epidemioloog afdeling medische besliskunde LUMC

drs V van der Meer, arts-onderzoeker afdeling medische besliskunde LUMC

**Correspondentie:**

dr JK (Jaap) Sont

Afd. Medisch Besliskunde, J-10-86

Leids Universitair Medisch Centrum

Albinusdreef 2

Postbus 9600

2300 RC Leiden

Tel: 071-5264578

Fax: 071-5266838

[J.K.Sont@lumc.nl](mailto:J.K.Sont@lumc.nl)

**SMASHING in adults**

**Self-Management of Asthma Supported by Hospitals, Information and communication technology, Nurses and General practitioners**

***Zelfmanagement bij volwassenen met astma ondersteund door ziekenhuizen, informatie en communicatie technologie, astmaverpleegkundigen en huisartsen***

**Summary**

Key words:

Asthma/*prevention & control/*economics/physiopathology/*therapy, Anti-Asthmatic Agents/*therapeutic use, Quality of Life, Self Care, Economics, Patient Education/*economics, Quality-Adjusted Life Years

### Summary (English and Dutch version): (no more than 50 lines)

*Objective*

1. To improve patient's quality of life in a cost-effective way by a self-management programme guided by doctors and a specialist asthma nurse through information and communication technology.

2. To investigate whether a simple index of asthma control and/or patient characteristics can be used to predict efficiency of such a guided self-management programme in order to target cost-effective implementation.

*Research Question*

1. A. What is the cost-effectiveness of a self-management programme in adult patients with mild to moderated asthma facilitated by information and communication technology and supported by health care professionals in order to improve quality of life? B. Does this self-management programme lead to improved asthma related quality of life?

2. Can the efficiency of such a guided self-management programme be predicted by a simple index of asthma control and/or patient characteristics and self-efficacy?

*Design*

A randomised parallel trial with 2 arms and 1 year follow-up in 2 phases in order to compare ICT-supported care with usual care. The first phase serves as a baseline period and is solely aimed at collecting data on asthma control and behaviour and lifestyle, and predisposing, reinforcing and enabling factors. The second phase includes the intervention and evaluation period.

*Study Population*

Patients with mild to moderate persistent asthma, aged between 18 and 50, will be identified via the LUMC general practice network (N=350) and hospital information systems (N=150). Patients have access to a computer with an internet-connection and are able to communicate in the Dutch language.

*Intervention*

The current intervention is additional to usual care and includes monitoring of symptoms and lung function and communication of results, feedback and reminders via internet and short message services on telephones. The service is supervised by a specialised nurse and facilitates discussion groups, a chat box and consultation via private messaging. Asthma self-management education and training sessions are given by a specialist nurse.

*Outcome measures*

The primary and secondary outcome are health related quality of life (AQLQ) and asthma control (ACQ), respectively.

*Sample size calculation*

Using 100 patients per arm and based on a SD of changes in AQLQ-score of 0.75 we are able to detect a difference of 0.3 points between AQLQ-changes of the two arms ( = 0.05 two-sided, ß = 0.20 one-sided).

*Economic evaluation*

The costs of usual care and ICT-supported care will be compared from a societal perspective. In a cost-utility analysis, the difference in societal costs will be related to the estimate difference in QALYs using acceptability curves. A Markov model will be used to extrapolate the trial data to a 3 year period.

**Samenvatting**

*Doel*

1. Het op kosten-effectieve wijze verbeteren van de kwaliteit van leven van volwassen met astma middels een zelfmanagementprogramma, waarbij patiënten ondersteunt worden door astmaverpleegkundigen, huisartsen, ziekenhuizen en informatie en communicatie technologie.

2. Onderzoeken in hoeverre een eenvoudige index van de mate van controle van het astma, naast patiëntenkarakteristieken, in staat is de efficiëntie van een dergelijk programma te voorspellen.

*Vraagstelling*

1. A. Wat is de kosten-effectiviteit van een programma gericht op het verbeteren van de kwaliteit van leven van volwassen met astma middels een zelfmanagementprogramma, waarbij patiënten ondersteunt worden door astmaverpleegkundigen, huisartsen, ziekenhuizen en informatie en communicatie technologie? B. Leidt dit zelfmanagementprogramma tot een verbeterde kwaliteit van leven?

2. In hoeverre kan een index van de mate van controle van het astma, naast patiëntenkenmerken, de efficiëntie van een dergelijk programma voorspellen?

*Studie-opzet*

Een gerandomiseerde parallelle trial met 2 armen en 1 jaar follow-up in 2 fasen, waarbij ICT ondersteunde zorg met de huidige zorg wordt vergeleken. De eerste fase dient als baseline periode, waarin gegevens omtrent de mate van controle van astma, kennis, eigen effectiviteit en gedrag en levensstijl zullen worden verzameld. De tweede fase betreft de interventie en evaluatie fase van de trial.

*Studie Populatie*

Patiënten met matig tot matig ernstig astma (behandelstap 2-4, leeftijd tussen 18 en 50) zullen worden gezocht in bestanden van het LUMC huisartsennetwerk (N=350) en ziekenhuis informatiesystemen (N=150). Patiënten hebben de beschikking over een computer met internetaansluiting en zijn in staat in de Nederlandse taal te communiceren.

*Interventie*

De te onderzoeken interventie is aanvullend aan de bestaande zorg bij astma en betreft ICT applicaties voor het monitoren van longfunctiemetingen - en klachten, 'feedback', en herinneringsboodschappen via internet en SMS berichten. Het geheel wordt gesuperviseerd door een astmaverpleegkundige en omvat tevens een internet discussie groep, 'chatbox' en consultaties via private messages. Astma zelfmanagement educatie en training zal door de verpleegkundige in kleine groepssessies worden gegeven.

*Uitkomstmaten*

De primaire en secundaire uitkomstmaten zijn, respectievelijk, de astma gerelateerde kwaliteit van leven (AQLQ) en de mate van controle van het astma (ACQ) .

*Sample size calculation*

Met 100 patiënten per behandelarm en gebaseerd op een SD van veranderingen in AQLQ-score van 0.75 zijn we in staat om een verschil van minstens 0.3 punten tussen de 2 armen te detecteren ( = 0.05 tweezijdig, ß = 0.20 eenzijdig).

*Economische evaluatie*

De kosten van de huidige zorg en ICT-ondersteunde zorg zullen worden vergeleken vanuit maatschappelijk perspectief. In een kosten-utiliteitsanalyse zullen de maatschappelijke kosten worden gerelateerd aan het de schattingen van het verschil in QALY's middels acceptabiliteitscurven. Met gebruikmaking van een Markov model worden de onderzoeksdata geëxtrapoleerd over 3 jaar.

**Principal Investigator**

Titles Dr

Initials J.K.

Prefix

Surname Sont male

Suffix

Phone number 071-5264578

Fax number 071-5266838

E-mail address j.k.sont@lumc.nl

Organisation Leiden University Medical Center

Suborganisation

Department Medical Decision Making

Postal address / nr. PO BOX 9600

Postal code / City 2300 RC City : Leiden

**Projectgroup**

Name Organisation Department

Dr J.K. Sont epidemiologist LUMC Medical Decison Making

Drs V. van der Meer research fellow LUMC Medical Decison Making

Dr H.F. van Stel epidemiologist LUMC Medical Decison Making

Dr H.A. Thiadens general practitioner LUMC General Practice

Dr E.H.D. Bel pulmonologist LUMC Pulmonology

Prof. Dr. W.J.J. Assendelft general practitioner LUMC General Practice

Prof. Dr. K.F. Rabe pulmonologist LUMC Pulmonology

Dr W. Otten psychologist LUMC Medical Decison Making

Prof. Dr A.A. Kaptein psychologist LUMC Medical Psychology

Dr W.B. van den Hout econometrist LUMC Medical Decison Making

Dr P.J. Toussaint informatics manager LUMC Medical Informatics

Prof. Dr P.J. Sterk respiratory physiologist LUMC Pulmonology

*Introduction*

Asthma is a chronic inflammatory disorder of the airways, which leads to recurrent episodes of wheezing, breathlessness, chest tightness and cough particularly at night and/or in the early morning. These symptoms are usually widespread and with variable airflow limitation that is at least partly reversible either spontaneously or with treatment. To date, asthma affects about 8% of the general population (1).

Due to the episodic nature of an asthma attack, individuals must maintain control of their disease with appropriate use of their medications and with proper self-management. Asthma guidelines emphasise that clinicians should teach patients about asthma, teach medication skills with aerosol devices, teach self-monitoring skills and environmental control/avoidance strategies. A Cochrane systematic review showed that asthma self-management education which consists of information, self-monitoring, regular medical review, and a written action plan is effective and leads to a reduction in hospitalisation and emergency room visits for asthma, days lost from work, episodes of nocturnal asthma, indirect costs and an improvement in quality of life (2). While a structured asthma self-management programme is effective in a hospital setting, attempts to deliver these programmes in primary care have met with varying success (3).

Indeed, despite the introduction of asthma guidelines the current level of asthma control in many patients falls far short of the goals for long-term asthma management. Patients tend to underestimate the severity of their condition and overestimate control, thereby tolerating, as an acceptable degree of control, a much higher rate of symptoms than recommended by medical professionals (4). Poor inhaler technique and repeated non-attendance for practice/hospital appointments are associated with an increased risk of asthma death (5). Each day, patients make decisions about taking medications, smoking, exposure to triggers, and participation in activities. The consequences of the choices patients make about asthma care accrue first and foremost to patients themselves. Ultimately, patients have the complete responsibility for the self-management of asthma.

However, the societal costs of asthma are considerable. Asthma negatively affects work productivity (6) as well as labour force participation (7). Furthermore, a recent survey showed that in the Netherlands 30% of asthmatics needed urgent care in the past year, which was significantly more than other European countries (8). Under a system designed for acute rather than chronic care, patients are not adequately taught to care for their own illness. Sixty-two percent of patients visit their pulmonary specialists or general practitioners only if they have an acute health problem (8). Only 15% of Dutch asthmatics had a doctor written action plan for asthma. In Australia, ownership of a written action plan seems to be falling (9). Physicians frequently complain about the lack of adequate time to spend with their patients to address all aspects of chronic asthma management. Patients will not cooperate with any intervention that is less than fully backed by the time and personal commitment of their doctors. Therefore, a major bottleneck in current asthma management concerns communication and participation (10).

It has been advocated that a partnership of patients and health care providers is needed to provide expertise related to the self-management of asthma, help patients acquire the knowledge necessary to make informed decisions about self-management, teach self-care techniques, provide social and emotional support, offer suggestions for behaviour change and coping strategies and create opportunities for patients to reflect on the choices they are making and the goals they hope to achieve. The American National Guidelines 2002 update recommends regular symptom and/or lung function peak-flow monitoring for patients with moderate to severe persistent asthma to provide a way to enhance communication between patients and clinicians and to increase patient awareness of their disease status and control, thereby helping patients ‘tune in’ to their disease (11). The need to show value to the patient and family is key to the use of self-assessment tools. Frequent feedback through communication between health care professional and patient may provide the best results on asthma management and control.

The safety and cost-effectiveness of asthma self-management education as such has been fully established nationally and internationally. In addition, there is increasing evidence that ICT can play a role in order to deliver components of these programmes in primary and secondary care. However, the current study is the first project in adult patients with asthma that integrates all 4 components of self-management education: information, self-monitoring, regular medical review and a written action plan. It is the first study that evaluates the cost-effectiveness of support by innovative information and communication technology.

*Hypothesis*

A self-management programme guided by doctors and a specialist asthma nurse through information and communication technology will improve asthma related quality of life in a cost-effective way.

*Objective*

1. To improve asthma related quality of life in a cost-effective way by a self-management programme guided by doctors and a specialist asthma nurse through information and communication technology.

2. To investigate whether a simple index of asthma control and/or patient characteristics can be used to predict efficiency of such a guided self-management programme in order to target cost-effective implementation.

*Research Questions*

1. A. What is the cost-effectiveness of a self-management programme in adult patients with mild to moderated asthma facilitated by information and communication technology and supported by health care professionals in order to improve quality of life in adults with asthma? B. Does this self-management programme lead to improved asthma related quality of life?

2. Can the efficiency of such a guided self-management programme be predicted by a simple index of asthma control and/or patient characteristics and self-efficacy?

*Study Population*

- 500 patients with mild to moderate persistent asthma (prevalent cases) will be identified via the registry of the LUMC general practice network (350 patients) and hospital information systems (150 patients: LUMC, Diaconessenhuis Leiden, and Leyenburg Ziekenhuis, The Hague). An expected response rate of 50% leads to the inclusion of 250 patients.
- age 18-50 yr, doctors diagnosis of asthma, asthma severity step 2-4, patients who need inhaled corticosteroids as controller medication
- PC with internet connection available
- able to communicate in the Dutch language

*Design (see flowchart)*

The study has been designed as a randomised parallel trial with 2 arms and 1 year follow-up in 2 phases in order to reduce the patients’ threshold for participation in a trial. The first phase serves as a baseline period and is solely aimed at collecting data on the patients’ degree of asthma control, behaviour and lifestyle, and predisposing, reinforcing and enabling factors according to the PRECEDE-PROCEED model for health promotion (12). This is followed by a second phase that includes the intervention and evaluation period. This two-phase design allows patients to become accustomed to participation in a self management programme. Furthermore, this gives us the opportunity to evaluate whether patient characteristics predict participation in this programme.

*Phase 1*

- Asthma Therapy Assessment Questionnaire (ATAQ) (13)
- introduction visit, assessment of baseline patient characteristics (see Instrument Table and Measurement Scheme)
- 2-week baseline period with weekly electronic registration of Asthma Control Questionnaire (ACQ) (14) via internet and daily registration of lung function via SMS or internet

*Phase 2*

- randomisation in 2 asthma management strategies, in blocks of 4 patients stratified on 2 classes of asthma control problems (ATAQ) and primary or secondary care
- 12-month intervention period where the patients receive either ‘usual care’ or ‘ICT-supported care’ guided by a specialised asthma nurse and doctor.
- after 3 and 12 months a 2-wk evaluation period with weekly electronic registration of Asthma Control Questionnaire (ACQ) (14) via internet and daily registration of lung function via SMS or internet +Asthma Quality of Life Questionnaire

*Care strategies*

1. *Usual care*

According to the Dutch GP guidelines, patients are invited to visit their general practitioner every 3 months in order to titrate medication to the lowest level that is needed to maintain control. This frequency can be lowered to 1-2 visits per year once control of asthma has been achieved (15). Thirty percent of general practices have nurse practitioners providing self-management education. Patients are referred to a chest physician if sufficient control is not achieved within 3 months. Exacerbations of asthma are treated by either chest physician and general practitioner.

- advise to visit to general practitioner or specialist to assess present situation
  - review medication devices technique and adherence
- issue and explain paper asthma action plan, monitoring of lung function with Piko-1 spirometer
  - plan next doctor visits as needed

1. *ICT-supported care*

- asthma self-management education in small groups (2x) by trained asthma specialist nurse (see below)
  - discussion of ACQ and ATAQ data in order to assess present situation and electronic asthma action plan
  - review medication devices technique and adherence
  - plan next doctor visits as needed
- at least 6 weeks monitoring of asthma control by lung function and ACQ with electronic feedback through webpages and/or SMS
- virtual consulting room with asthma nurse via and private messaging
- social support within a private chatbox and/or internet support group
- automated sending of reminders via email and/or SMS
- monitoring asthma control by lung function and ACQ with electronic data processing and feedback through computer via webpages with graphical presentation of data for patient and nurse

*Asthma self-management education sessions*

The sessions will be organised along the lines of self-management education as applied in diabetes care (16) and is patient centred rather than provider centred. Information about asthma self-management is presented in response to questions from participants rather than in lectures. The education is focussed around meeting the patients’ needs and responding to their identified concerns. Patients work toward self-selected goals and are offered the option of follow-up through an internet-based support group and email contact with the specialist nurse. Follow-up is focussed on ongoing goal setting and behaviour change efforts and problem-solving (17).

*Internet applications*

- web application for monitoring of lung function and ACQ with support for receiving SMS messages (baseline and evaluation period)
- facility for sending reminders to stimulate monitoring via email and/or SMS
- patient portal for ICT-supported care
- monitoring of lung function and ACQ with processed data presentation and feedback via webpage or SMS
- facility for sending reminders to stimulate monitoring via email and/or SMS
- chatbox and internet support group facilities
- asthma education webpages
- private message contact with asthma specialist nurse:
  - design asthma action plan
  - goal-setting and registration of goal-attainment
- nurse portal to support patient self-management and review patient data

*Instruments (see Instrument table and Measurement scheme)*

- Asthma Therapy Assessment Questionnaire (ATAQ) (13)
- Asthma Control Questionnaire (ACQ), includes assessment of lung function (categories of FEV1 as % predicted) (14)
- Rosenberg Self-Esteem Scale (RSES) (18)
- Illness perception questionnaire (IPQ) (19)
- Intention/Willingness questionnaire (I/W) (20)
- Life Orientation Test (LOT) (21)
- Social Norm (SN) (22)
- Self-Reported Asthma Management Behaviour (SRAMB)
- Social Support List (SSL) (23)
- Patient Skills Scoring by Nurse (PSSN) (24)
- Asthma Quality of Life Questionnaire (AQLQ) (25)
- Knowledge, Attitude and Self-efficacy Asthma Questionnaire (KASE-AQ) (26)
- EQ-5D (27)
- Asthma Symptom Utility Index (ASUI) (24)

*Outcome parameters*

The choice of outcome parameters has been based on a recent review on asthma adherence intervention research that recommended to include disease-focused as well as patient-focused outcome measures besides adherence measures (28). Behaviour change is an important objective, however, it might be inadequate to establish benefit from an adherence intervention. Therefore, the primary and secondary outcome in this project are health related quality of life and asthma control, respectively.

*Prediction model*

In order to predict the efficiency of the ICT-supported self-management programme we will assess the relationship between potential predictors and change in quality of life using multiple linear regression analysis. The following baseline variables and patient characteristics will be included in the model: an index of asthma control (ATAQ), knowledge, attitudes, self-efficacy, self-esteem, illness perception, optimism, social norm, social support and intentions/willingness (listed under 'predisposing factors' in the table on page 18). The number of variables comprises approximately 10% of the number of patients in the intervention group.

*Sample size calculation*

Juniper et al. showed that the AQLQ had good evaluative properties with respect to a clinicians rating of a change in asthma control (29). Theoretically, the AQLQ-score ranges between 0 and 6 points. Using 100 patients per arm and a SD of changes in AQLQ-score of 0.75 (30) we are able to detect a difference of 0.3 points between AQLQ-changes of the two arms ( = 0.05 two-sided, ß = 0.20 one-sided). This is similar as the mean difference in quality of life between self-management education programmes and usual care that was reported in a recent Cochrane systematic review (31). A minimally important change in AQLQ score has been defined as 0.5 point (29). Such a change can be detected between the arms with 36 patients per group. Therefore, the current study has sufficient power to detect minimally important between-group differences in quality of life, even in a subgroup analysis for patients in a primary care as well as in a secondary care setting. The first phase allows patients to become accustomed to participation in a self management programme. We aim to identify 500 eligible patients. With an expected response rate of 50% 250 patients can be included. With respect to asthma control, using 100 patients per arm and a SD of changes in ACQ-score of 0.69 (32) we are able to detect a difference of 0.28 points between ACQ-changes of the two arms.

*Data-analysis and presentation / synthesis*

All analyses will be carried out on an intention to treat basis.

***Economic evaluation***

*General considerations*

The costs of usual care and ICT-supported care will be compared from a societal perspective. In a cost-utility analysis, the difference in societal costs will be related to the estimate difference in QALYs using acceptability curves. A Markov model will be used to extrapolate the trial data to a 3 year period (33;34).

The model will include data on asthma control, intervention efficacy, compliance, symptoms, acute exacerbations, QALYs, medication, implementation and other health care costs, productivity costs, and discounting. Uncertainty analysis will be carried out using Monte Carlo simulation techniques. Local and global (probabilistic) sensitivity analyses will be performed in order to address the generalisability of the results to different countries and settings with respect to population, patient characteristics, disease severity and patient adherence.

During the trial, health care consumption and absenteeism will be assessed every 3 months, using a cost questionnaire filled-out by the patient (35). Purchased medication will be retrieved from the patient's main pharmacist (with written patient permission), complemented with the patient's report on medication purchased elsewhere (36).

*Cost analysis*

A cost price analysis will be performed to evaluate the programme implementation costs (including costs for ICT development, asthma nurse time, patient time and travel costs, and costs of internet, chatbox, discussion group and SMS). Other health care consumption and absenteeism will be valued at standard prices or charges (37;38).

*Patient outcome analysis*

*Measurement of adherence*

- Adherence to lung function monitoring will be based on automated registration of values and date/time in the memory of the Piko-1 spirometer that can be uploaded to a personal computer and compared with the data entered by the patient via internet or SMS
- SMS-usage and usage of webpages will be documented and patients will be asked for the reason(s) in case of discontinuation of internet- and/or SMS-usage
- Self-management behaviour will be assessed by the SRAMB (see Instrument table)

*Measurement of effectiveness*

Effectiveness will be assessed at baseline, after 3 months and after 12 months of follow-up

- In order to calculate QALYs, preference based patient utilities will be assessed via EQ-5D and asthma symptom utility index (27) (24)
- quality of life: Asthma Quality of Life Questionnaire (AQLQ) (25)
- asthma control: Asthma Control Questionnaire (ACQ) (14)
- the number of limited activity days (39)
- lung function level and variability as assessed by the Piko-1 spirometer
- exhaled nitric oxide (40)

**References**

(1) Rijcken B, Kerkhof M, de Graaf A, Boezen HM, Droste JJ, Kremer AM. Europees Luchtweg Onderzoek Nederland. Rijksuniversiteit Goningen Epidemiologie 1996.

(2) Gibson PG, Coughlan J, Wilson AJ, Abramson M, Bauman A, Hensley MJ et al. Self-management education and regular practitioner review for adults with asthma. Cochrane Database Syst Rev 2000;CD001117.

(3) Gibson PG, Ram FS, Powell H. Asthma education. Respir Med 2003; 97:1036-1044.

(4) Rabe KF, Vermeire PA, Soriano JB, Maier WC. Clinical management of asthma in 1999: the Asthma Insights and Reality in Europe (AIRE) study. Eur Respir J 2000; 16:802-807.

(5) Sturdy PM, Victor CR, Anderson HR, Bland JM, Butland BK, Harrison BD et al. Psychological, social and health behaviour risk factors for deaths certified as asthma: a national case-control study. Thorax 2002; 57:1034-1039.

(6) Schermer TR, Thoonen BP, van den BG, Akkermans RP, Grol RP, Folgering HT et al. Randomized controlled economic evaluation of asthma self-management in primary health care. Am J Respir Crit Care Med 2002; 166:1062-1072.

(7) Blanc PD, Trupin L, Eisner M, Earnest G, Katz PP, Israel L et al. The work impact of asthma and rhinitis: findings from a population-based survey. J Clin Epidemiol 2001; 54:610-618.

(8) Vermeire PA, Rabe KF, Soriano JB, Maier WC. Asthma control and differences in management practices across seven European countries. Respir Med 2002; 96:142-149.

(9) Walters EH, Walters JA, Wood-Baker R. Why have asthma action plans failed the consumer test? Med J Aust 2003; 178:477-478.

(10) Partridge MR, Hill SR. Enhancing care for people with asthma: the role of communication, education, training and self-management. 1998 World Asthma Meeting Education and Delivery of Care Working Group. Eur Respir J 2000; 16:333-348.

(11) National Asthma Education and Prevention Program. Expert Panel Report: Guidelines for the Diagnosis and Management of Asthma Update on Selected Topics--2002. J Allergy Clin Immunol 2002; 110:S141-S219.

(12) Green LW, Kreuter MW. Health promotion planning: An educational and environmental approach. 2nd ed. Mayfield: Mountain View, Cal., 1991.

(13) Vollmer WM, Markson LE, O'Connor E, Frazier EA, Berger M, Buist AS. Association of asthma control with health care utilization: a prospective evaluation. Am J Respir Crit Care Med 2002; 165:195-199.

(14) Juniper EF, O'Byrne PM, Guyatt GH, Ferrie PJ, King DR. Development and validation of a questionnaire to measure asthma control. Eur Respir J 1999; 14:902-907.

(15) Folmer H, Smeenk FW, Geijer RM, van Hensbergen W, Molema J, Smeele IJ et al. Landelijke transmurale afspraak: astma bij volwassenen. Huisarts Wet 2001; 44(4):165-169.

(16) Funnell MM, Anderson RM. Working toward the next generation of diabetes self-management education. Am J Prev Med 2002; 22:3-5.

(17) Lorig K, Holman H, Sobel D, Laurent D, Gonzalez V, Minor M. Living a healthy life with chronic conditions. Self-management of heart disease, arthritis, diabetes, asthma, bronchitis, emphysema & others. 2nd ed. Boulder, CO: Bull Publishing Company, 2000.

(18) Rosenberg M. Society and the adolescent self-image. Princeton, New Jersey: Princeton University Press, 1965.

(19) Moss-Morris R, Weinman J, Petrie KJ, Horne R, Cameron LD, Buick D. The revised illness perception questionnaire (IPQ-R). Psychol Health 2002; 17(1):1-16.

(20) Gibbons FX, Gerrard M, Blanton H, Russell DW. Reasoned action and social reaction: willingness and intention as independent predictors of health risk. J Pers Soc Psychol 1998; 74:1164-1180.

(21) Scheier MF, Carver CS. Optimism, coping, and health: assessment and implications of generalized outcome expectancies. Health Psychol 1985; 4:219-247.

(22) Fishbein M, Ajzen J. Belief, attitude, intention and behaviour: An introduction to theory and research. Reading, MA: Addison-Wesley, 1975.

(23) Tempelaar R, de Haes JC, De Ruiter JH, Bakker D, Van Den Heuvel WJ, Van Nieuwenhuijzen MG. The social experiences of cancer patients under treatment: a comparative study. Soc Sci Med 1989; 29:635-642.

(24) Revicki DA, Leidy NK, Brennan-Diemer F, Sorensen S, Togias A. Integrating patient preferences into health outcomes assessment: the multiattribute Asthma Symptom Utility Index. Chest 1998; 114:998-1007.

(25) Juniper EF, Guyatt GH, Ferrie PJ, Griffith LE. Measuring quality of life in asthma. Am Rev Respir Dis 1993; 147:832-838.

(26) Wigal JK, Stout C, Brandon M, Winder JA, McConnaughy K, Creer TL et al. The Knowledge, Attitude, and Self-Efficacy Asthma Questionnaire. Chest 1993; 104:1144-1148.

(27) Szende A, Svensson K, Stahl E, Meszaros A, Berta GY. Psychometric and utility-based measures of health status of asthmatic patients with different disease control level. Pharmacoeconomics 2004; 22:537-547.

(28) Bender B, Milgrom H, Apter A. Adherence intervention research: what have we learned and what do we do next? J Allergy Clin Immunol 2003; 112:489-494.

(29) Juniper EF, Guyatt GH, Willan A, Griffith LE. Determining a minimal important change in a disease-specific quality of life questionnaire. J Clin Epidemiol 1994; 1:81-87.

(30) Thoonen BP, Schermer TR, van den BG, Molema J, Folgering H, Akkermans RP et al. Self-management of asthma in general practice, asthma control and quality of life: a randomised controlled trial. Thorax 2003; 58:30-36.

(31) Gibson PG, Powell H, Coughlan J, Wilson AJ, Abramson M, Haywood P et al. Self-management education and regular practitioner review for adults with asthma (Cochrane Review). Cochrane Database Syst Rev 2003;CD001117.

(32) Juniper EF, O'Byrne PM, Roberts JN. Measuring asthma control in group studies: do we need airway calibre and rescue beta2-agonist use? Respir Med 2001; 95:319-323.

(33) Price MJ, Briggs AH. Development of an economic model to assess the cost effectiveness of asthma management strategies. Pharmacoeconomics 2002; 20:183-194.

(34) Van Den Brink M, Van Den Hout WB, Stiggelbout AM, Klein KE, Marijnen CA, Van De Velde CJ et al. Cost-utility analysis of preoperative radiotherapy in patients with rectal cancer undergoing total mesorectal excision: a study of the Dutch Colorectal Cancer Group. J Clin Oncol 2004; 22:244-253.

(35) Van Den Hout WB, Tijhuis GJ, Hazes JM, Breedveld FC, Vliet Vlieland TP. Cost effectiveness and cost utility analysis of multidisciplinary care in patients with rheumatoid arthritis: a randomised comparison of clinical nurse specialist care, inpatient team care, and day patient team care. Ann Rheum Dis 2003; 62:308-315.

(36) Van Den Brink M, Van Den Hout WB, Stiggelbout AM, Van De Velde CJ, Kievit J. Cost measurement in economic evaluations of health care: Whom to ask? Med Care 2004; 42:740-746..

(37) Oostenbrink JB, Koopmanschap MA, Rutten FF. Standardisation of costs: the Dutch Manual for Costing in economic evaluations. Pharmacoeconomics 2002; 20:443-454.

(38) Dutch Health Insurance Executive Board. Pharmacotherapeutic Compass. 2003 (in Dutch) ed. Amstelveen: Dutch Health Insurance Executive Board, 2003.

(39) Dennis SM, Altmann DR, Lee TH. Increase in daytime symptoms is a sensitive and specific criterion for predicting corticosteroid-treated exacerbations in a clinical asthma trial. Clin Exp Allergy 2005; 35:308-312.

(40) Ricciardolo FL, Sterk PJ, Gaston B, Folkerts G. Nitric oxide in health and disease of the respiratory system. Physiol Rev 2004; 84:731-765.

Bijlage: flowchart

**Bijlage: instrument table and measurement scheme**

|  | SRAMB | KASE | Social norm | Social Support List | Intentions/Willingnes | IPQ | LoT | RSES | ACQ | ATAQ | AQLQ | PSSN | LF + daily sympoms | FeNO | Cyranose 320 | (Cost) Questionnaire | ASUI | EQ-5D |
| --- | --- | --- | --- | --- | --- | --- | --- | --- | --- | --- | --- | --- | --- | --- | --- | --- | --- | --- |
| **Predisposing factors** |  |  |  |  |  |  |  |  |  |  |  |  |  |  |  |  |  |  |
| Knowledge  Attitudes  Self-efficacy  Self-esteem  Illness Perception  Optimism  Social Norm  Social Support  Intentions/Willingness |  | X  X  X | X | X | X | X | X | X |  | X  X |  |  |  |  |  |  |  |  |
| **Reinforcing factors** |  |  |  |  |  |  |  |  |  |  |  |  |  |  |  |  |  |  |
| Patient/provider communucat.  Attitude/behaviour environm.  Attitude/behaviour personnel |  | X  X  X | X |  |  |  |  |  |  | X |  |  |  |  |  |  |  |  |
| **Enabling factors** |  |  |  |  |  |  |  |  |  |  |  |  |  |  |  |  |  |  |
| Availability of resources  Education required  Skills (correct use inhaler) |  |  |  |  |  |  |  |  |  | X  X |  | X |  |  |  |  |  |  |
| **Behaviour** |  |  |  |  |  |  |  |  |  |  |  |  |  |  |  |  |  |  |
| Preventive actions  Compliance medication  Compliance monitoring FEV1  Management of exacerbations | X  X  X  X |  |  |  |  |  |  |  |  |  |  |  | X |  |  |  |  |  |
| **Asthma control** |  |  |  |  |  |  |  |  |  |  |  |  |  |  |  |  |  |  |
| Symptom severity  β2-agonist usage  Airway obstruction |  |  |  |  |  |  |  |  | X  X | X  X |  |  | X |  |  |  |  |  |
| **Disease markers** |  |  |  |  |  |  |  |  |  |  |  |  |  |  |  |  |  |  |
| Exhaled nitric oxide  Smell print |  |  |  |  |  |  |  |  |  |  |  |  |  | X | X |  |  |  |
| **Outcomes** |  |  |  |  |  |  |  |  |  |  |  |  |  |  |  |  |  |  |
| Asthma related quality of life  Asthma control  Symptom free days  Limited activity days  Days off work  Exacerbations  No. of prednison courses  Costs  Utility |  |  |  |  |  |  |  |  | X |  | X |  | X  X |  |  | X  X  X  X | X | X |

SRAMB= self reported asthma management behaviour questionnaire

KASE=knowledge, attitude and self-efficacy of asthma questionnaire

IPQ= illness perception questionnaire

LoT= life orientation test

RSES= Rosenberg self-esteem scale

ACQ=asthma controle questionnaire

ATAQ=asthma therapy assessment questionnaire

AQLQ=asthma quality of life questionnaire

PSSN= practical skills scored by nurse

LF= lung function

FeNO= exhaled nitric oxide

Cyranose 320= chemical vapor analyzer, including a nanocomposite sensor array with 32 polymer sensors (zie protocolaanvraag 05/119)

ASUI= asthma symptom utility index

**measurement scheme**

| **instrument** | items | version available | baseline | t=12 | t=26 | t=39 | t=52 |
| --- | --- | --- | --- | --- | --- | --- | --- |
| Demography |  | UC* | X |  |  |  |  |
| SRAMB** | 45 | UC* | X X | X |  |  | X |
| KASE | 60 | NL | X |  |  |  | X |
| Social norm | 28 | NL | X |  |  |  |  |
| Social Support | 16 | NL | X |  |  |  |  |
| Intention/Willingness | 6 | NL | X |  |  |  |  |
| IPQ | 9 | NL | X |  |  |  |  |
| LoT | 10 | NL | X |  |  |  |  |
| RSES | 10 | NL | X |  |  |  |  |
| ACQ¶ | 7 | NL | X | X | X | X | X |
| ATAQ | 14 | NL | X | X |  |  | X |
| AQLQ | 32 | NL | X | X |  |  | X |
| PSSN | 1 |  |  |  |  |  |  |
| LF + daily symp¶¶ | 3 | NL | X | X |  |  | X |
| FeNO | 1 | nvt | X |  |  |  | X |
| Cyranose 320 | 1 | nvt | X |  |  |  |  |
| (Cost) questionnaire |  | UC* | X | X | X | X | X |
| ASUI | 11 | English | X | X |  |  | X |
| EQ-5D | 5 | NL | X |  |  |  | X |

UC* = under construction

SRAMB**: at start and end of baseline to assess test-retest reliability

¶ = ACQ: intervention group: weekly

control group: at baseline, t=12 and t=52

¶¶ = LF + daily symptoms:

intervention group: baseline until t=6, t=10-12, t=50-52

control group: baseline (2 weeks), t=10-12, t=50-52
