# Supplementary figures and images for: Cost-Effectiveness of Internet-Based Self-Management Compared with Usual Care in Asthma
Source: PLoS One. 2011 Nov 11;6(11):e27108. doi: 10.1371/journal.pone.0027108 (PMC3214043; doi:10.1371/journal.pone.0027108)

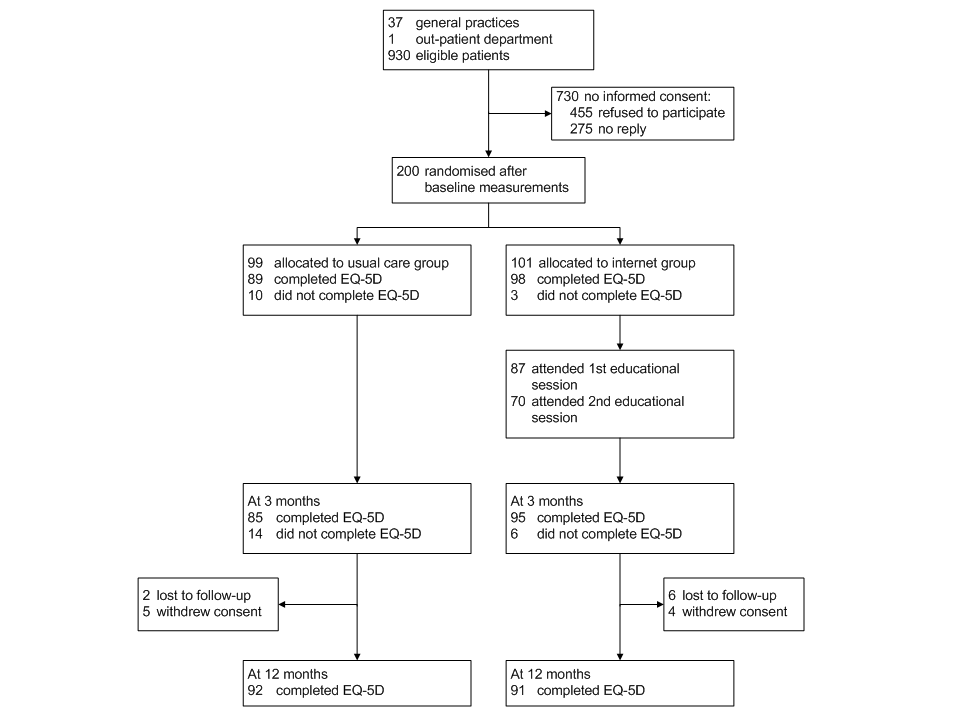

Supplement: Flowchart S1 — CONSORT flowchart (TIF) [file pone.0027108.s003.tif]
